# Modest changes in *Spi1* dosage reveal the potential for altered microglial function as seen in Alzheimer’s disease

**DOI:** 10.1038/s41598-021-94324-z

**Published:** 2021-07-22

**Authors:** Ruth E. Jones, Robert Andrews, Peter Holmans, Matthew Hill, Philip R. Taylor

**Affiliations:** 1 Division of Infection and Immunity, Cardiff University, Cardiff, UK; 2 UK Dementia Research Institute at Cardiff, Cardiff University, Hadyn Ellis Building, Maindy Road, Cardiff, CF24 4HQ UK; 3 Division of Psychological Medicine and Clinical Neurosciences, Cardiff University, Cardiff, UK; 4 Systems Immunity Research Institute, Cardiff University, Cardiff, UK

**Keywords:** Inflammation, Monocytes and macrophages, Neuroimmunology, Alzheimer's disease

## Abstract

Genetic association studies have identified multiple variants at the *SPI1* locus that modify risk and age of onset for Alzheimer’s Disease (AD). Reports linking risk variants to gene expression suggest that variants denoting higher *SPI1* expression are likely to have an earlier AD onset, and several other AD risk genes contain PU.1 binding sites in the promoter region. Overall, this suggests the level of *SPI1* may alter microglial phenotype potentially impacting AD. This study determined how the microglial transcriptome was altered following modest changes to *Spi1* expression in primary mouse microglia. RNA-sequencing was performed on microglia with reduced or increased *Spi1*/PU.1 expression to provide an unbiased approach to determine transcriptomic changes affected by *Spi1.* In summary, a reduction in microglial *Spi1* resulted in the dysregulation of transcripts encoding proteins involved in DNA replication pathways while an increased *Spi1* results in an upregulation of genes associated with immune response pathways. Additionally, a subset of 194 *Spi1* dose-sensitive genes was identified and pathway analysis suggests that several innate immune and interferon response pathways are impacted by the concentration of *Spi1*. Together these results suggest *Spi1* levels can alter the microglial transcriptome and suggests interferon pathways may be altered in individuals with AD related *Spi1* risk SNPs.

## Introduction

Alzheimer’s Disease (AD) is the most prevalent form of Dementia, affecting millions of people worldwide^1^. Studies investigating AD genetics and pathology have suggested immune gene networks may contribute to an increased risk of developing AD^2,3^. *SPI1* encodes PU.1, a central transcription factor in microglial development and activation, and has a genome-wide significant genic association with AD in the IGAP GWAS (rs3740688 Odds Ratio 0.92 Meta p = 5.4 × 10^−13^), comprising 35,274 Alzheimer’s disease cases and 59,163 controls^4^. In addition, a 56-protein interaction network consisting of strongly enriched rare coding variants (p = 1.08 × 10^–7^, and common variants with Late-Onset AD gene-wide significance (p = 2.98 × 10^–7^) identified *SPI1* as a central hub gene^5^. Several studies have suggested *SPI1*/PU.1 levels impact on the microglial transcriptome, therefore affecting the phenotype of these cells. Several single-nucleotide polymorphisms (SNPs) associated with an increased risk of AD are thought to lie within the *Spi1* gene locus^3,6,7^. The SNP variant rs1057233^a^ is thought to result in a higher level of *Spi1* expression and earlier age of AD onset^7^. Moreover, *Spi1* is thought to influence the expression of other AD risk genes^5,7,8^.

The PU.1 transcription factor is essential for the survival and function of macrophages^9–13^ and is well conserved between humans and mice (> 85% protein similarity, BLAST protein alignment (RefSeq ID’s NP_003111.2 and NP_035485, respectively). In hematopoietic development low levels of PU.1 drives B-lymphocyte development whereas cells expressing high levels of PU.1 are committed to the myeloid lineage^14–16^. In development PU.1 levels are regulated to commit precursors to a macrophage or B-cell lineage^17–23^. In these early experiments PU.1 appeared to have dose-dependent transcriptional thresholds in foetal liver macrophages^24^. PU.1 also regulates expression of several key macrophage receptors such as CSF1R, CD11b and CD45^25–27^. Moreover PU.1 interacts with other lineage-determining factors such as C/EPBα/β to alter the chromatin landscape resulting in a specialised macrophage epigenome^28–30^.

In both primary human microglia and the BV-2 mouse microglia cell line, reductions in PU.1 have resulted in changes to gene expression and a reduced phagocytic capacity^7,31,32^. Additionally, increased PU.1 expression in BV-2 cells resulted in increased zymosan phagocytosis, and amplified both ROS, NO and cytokine production after LPS stimulation^32^. Though the impact of altered *Spi1* on the microglial transcriptome would ideally be studied in freshly isolated cells, such as those from a transgenic mouse model, at the time of this study no appropriate *Spi1* over-expression transgenic mouse lines were available.

CSF1R inhibitors are a potential AD therapeutic and prevent AD-associated microgliosis by blocking the CSF1R/PU.1 survival signalling pathway^33–36^ but the impact on peripheral macrophages has not been reported. Knowing how subtle changes in *SPI1*/PU.1 levels contribute to the microglial tissue resident subset transcriptome could allow more specific pathways could be targeted.

RNA-sequencing was used to assess changes to the microglial transcriptome following modest changes to *Spi1* expression in microglia from primary mixed-glia cultures. Modest changes to PU.1 protein were desirable as they reflect the expression changes caused by the risk allele, and therefore the biology underlying AD. A moderate reduction in microglial *Spi1* resulted in altered expression of cell cycle related genes while small *Spi1* increases upregulated immune response genes. A subset of genes identified as *Spi1* dose-sensitive highlighted a potential dose-dependent interferon driven immune response regulated by *Spi1*.

## Results

### RNA-sequencing data shows impact of *Spi1* dose on microglia transcriptome

**Figure 1:**
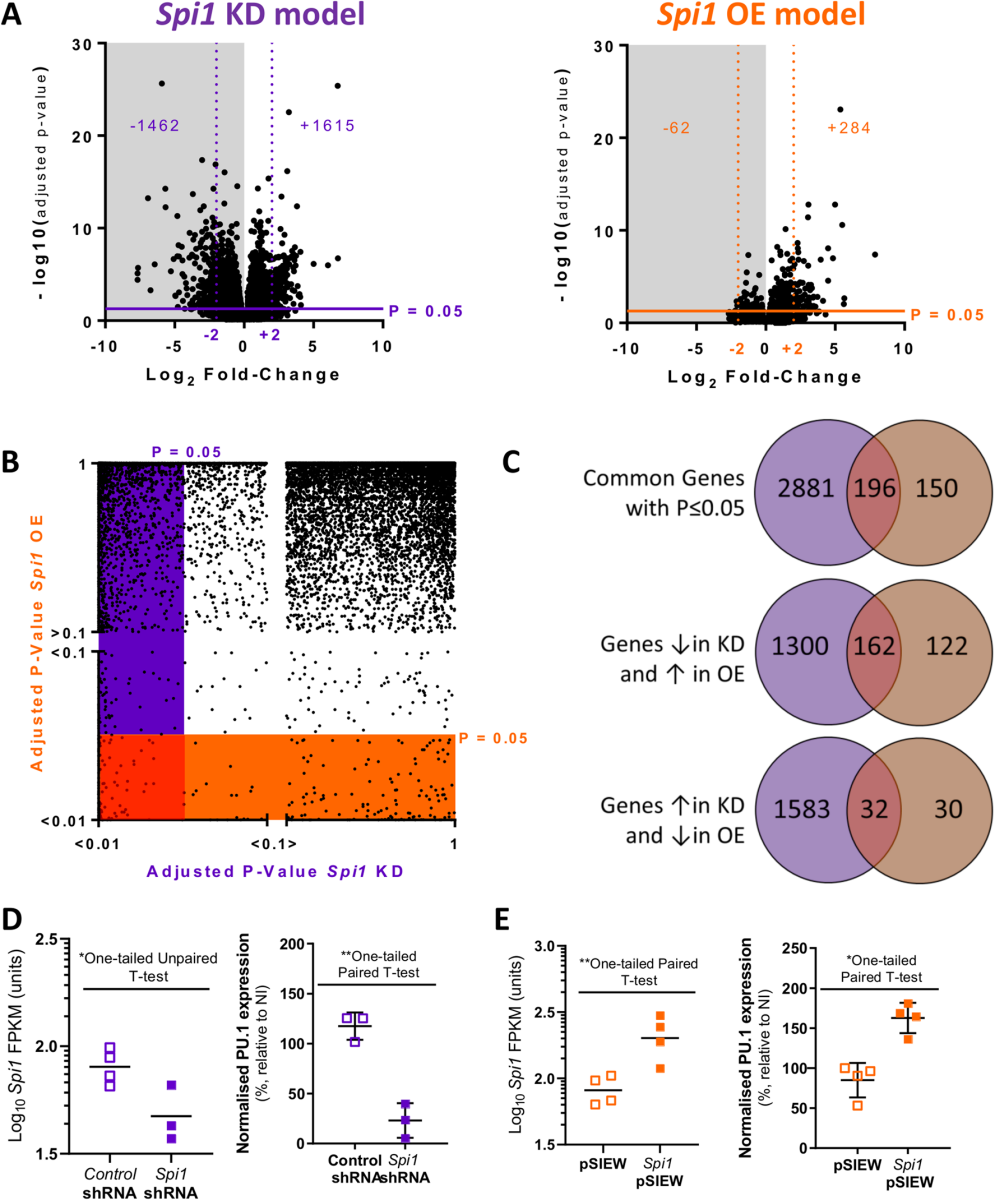
Results of RNA-Seq experiment in primary mixed-glial cultures (**A**) Volcano plots summarising the distribution of genes in the *Spi1* knock-down (purple) and *Spi1* over-expression (orange). In the *Spi1* knock-down dataset a 1462 genes were down-regulated (grey background) and 1615 up-regulated with a *p* value of ≤ 0.05 (solid lines), as indicated by the numbers on the graph. In the *Spi1* over-expression dataset the majority of the genes, 284, were up-regulated and 62 genes had down-regulated expression. (**B**) Genes that were significantly changed in both the *Spi1* knock down (KD, purple) and the *Spi1* over expression (OE, orange) using a *p* ≤ 0.05 threshold. A Plot of adjusted *p* values from all genes in both datasets. Genes that were below the *p* ≤ 0.05 threshold in the *Spi1* knock-down dataset are highlighted in purple, those that were below the *p* ≤ 0.05 cut-off in the *Spi1* over-expression are within the orange bar. In the bottom left corner the red line surrounds the 196 genes that were significantly changed in both datasets. (**C**) Venn diagrams summarising the gene expression that were significantly changed (*p* ≤ 0.05) in either the *Spi1* knock-down dataset (purple), the *Spi1* over-expression dataset (orange) or changed in both datasets (red). Of interest were the genes with expression that appears to be sensitive to the *Spi1* dose in microglia, namely the 194 genes that appear to be expressed relative to the dose of *Spi1*. (**D**) The log_10_ number of *Spi1* mRNA fragments per Kilobase of transcript per Million mapped reads (FPKM) shows that *Spi1* was lower in *Spi1* shRNA infected microglia than in control shRNA infected microglia (One-tailed Unpaired T-test on the log_10_ transformed data, *p* = 0.0166, n = 4 for control shRNA and n = 3 for *Spi1* shRNA). PU.1 protein expression was normalised to the non-infected (NI) samples in each experiment (as described in methods). Microglia infected with the *Spi1* shRNA (solid purple) have reduced PU.1 expression compared to cells infected with a control shRNA (lined purple) (One-tailed Paired T-test, *p* = 0.0013, n = 3 per group). (**E**) *Spi1* mRNA expression (FPKM) was increased in the samples infected with the *Spi1* over-expression construct (filled orange) compared to empty vector control samples (outline orange), (One-tailed Paired T-test on the Log_10_ transformed data, *p* = 0.0013, n = 4 per group).The *Spi1* over-expression virus (*Spi1* pSIEW) increased PU.1 protein expression in microglia by roughly half compared to cells infected with an empty vector control (lined orange). One-tailed Paired T-test, *p* = 0.0131, n = 4 per group) (**D**–**E**) Each dot represents the value from one biological replicate, the means are indicated by the horizontal lines and the error bars display the standard deviation about the mean. All experiments were performed using mixed glia cultures from 8-week-old female C57BL/6J mice. Figures (**A**–**C**) were made in GraphPad PRISM 6 (version 3.07) and (**D**–**E**) in GraphPad PRISM 8 (version 8.4.3; both GraphPad Software, Inc.).

The effect of *Spi1* shRNA knock down (KD) and *Spi1* pSIEW over expression (OE) was assessed in flow cytometric sorted microglia after 11 days of lentivirus infection. Following *Spi1* knock-down (*Spi1* shRNA compared to control shRNA) 1615 genes were up- and 1462 down-regulated, using an adjusted *p* value of < 0.05 cut-off (Benjamini–Hochberg corrected for multiple testing). A proportion of down-regulated genes (230 genes) surpassed the -2 log_2_ fold-change cut-off (Fig. 1A). In the *Spi1* over-expression dataset (*Spi1* pSIEW compared to pSIEW) 284 genes had an increased expression, 62 were down-regulated (*p* < 0.05). In this comparison 71 up-regulated and 4 down-regulated exceeded a log_2_ fold-change value of 2. Principal component analysis (PCA, Supplementary Fig. 1) confirmed that samples within the same group had greater similarity, while the *Spi1* pSIEW and *Spi1* shRNA samples clustered separately.

The datasets with altered *Spi1* expression were then compared to identify which subset of genes were likely affected by *Spi1*/PU.1 in a dose-dependent manner. This identified 196 genes which were significantly diminished in the *Spi1* knock-down and upregulated in the *Spi1* over-expression dataset (Adjusted *p* value of ≤ 0.05; Fig. 1B). When the direction of the gene fold-changes was compared 162 of these genes were lower in the dataset with lower *Spi1* and higher in the *Spi1* over-expression dataset and 32 genes where expression was increased following *Spi1* knock-down and reduced in the *Spi1* over-expression dataset (Fig. 1C). Therefore, 194 genes were classed as *Spi1* dose-sensitive (Supplementary Table 1).

RNA-sequencing confirmed *Spi1* fragments per Kilobase of transcript per Million mapped reads (FPKM) values were significantly lower in cultures infected with *Spi1* shRNA as opposed to the control shRNA (Fig. 1D, One-Tailed Unpaired T-test on Log_10_ data *p* = 0.0166). *Spi1* mRNA expression was increased in the *Spi1* pSIEW samples compared to the control virus (Fig. 1E, One-Tailed Paired T-test on Log_10_ data *p* = 0.0013).

PU.1 protein was assessed in independent microglia samples (Fig. 1D–E) and was reduced by ~ 70–80% in *Spi1* shRNA samples compared to control shRNA infected microglia (Fig. 1D One-tailed Paired T-test, *p* = 0.0013 and Supplementary Fig. 3B One-tailed Paired T-test, *p* = 0.001). *Spi1* over-expression in microglia increased PU.1 protein expression by ~ 70% compared to microglia infected with pSIEW control virus (One-tailed Paired T-test, *p* = 0.0131). The values clearly cluster within each biological group and the means of *Spi1* shRNA and *Spi1* pSIEW microglia compared to the appropriate control viruses are disparate indicating PU.1 was altered by these *Spi1* modulating viruses.

### Gene ontology analysis in *Spi1* knock-down and *Spi1* over-expression datasets

**Figure 2:**
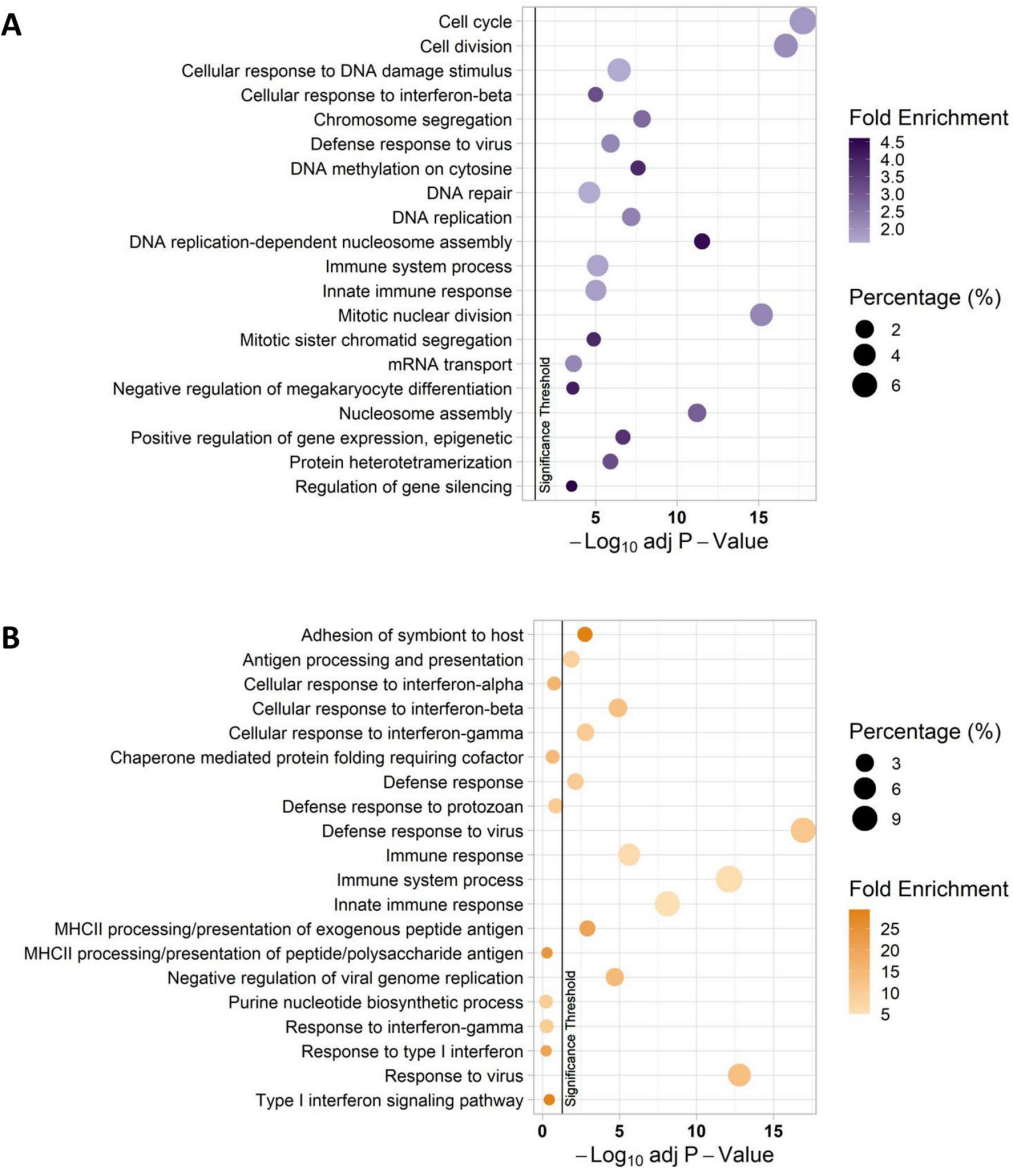
The 20 most siginificantly changed biological pathways in the *Spi1* knock-down (**A**) and *Spi1* over-expression (**B**) datasets assessed using DAVID. In these graphs the Benjamini–Hochberg adjusted *p* value(− Log_10_) is displayed on the x-axis, the Gene Ontology (GO) term listed on the y-axis, the percentage of the gene list in each cluster is denoted by the size of the bubble and the colour denotes the fold-change, where a darker colour indicates a stronger enrichment. The vertical black line indicates the *p* value cut-off of 0.05. Bubble plots were made using the ‘tidyverse’^37^ and ‘ggpubr’^38^ packages in R^39,40^.

Differentially expressed genes (*p* < 0.05 significance threshold) in the *Spi1* KD and *Spi1* OE datasets were separately assessed using DAVID (Database for Annotation, Visualization and Integrated Discovery, version 6.8). Figure 2 displays the Gene Ontology (GO) terms from the 20 most significantly enriched pathways in each dataset (corrected *p* values *p* < 0.05, FDR). In primary microglia with a lower *Spi1* expression the most significantly changed pathways were related to cell cycle and DNA repair (Fig. 2A). However, microglia with higher *Spi1* (Fig. 2B) had an over representation of genes related to antigen presentation pathways, immune system processes and response to interferon.

### *Spi1* dose-sensitive subset highlight interferon response pathways

**Figure 3:**
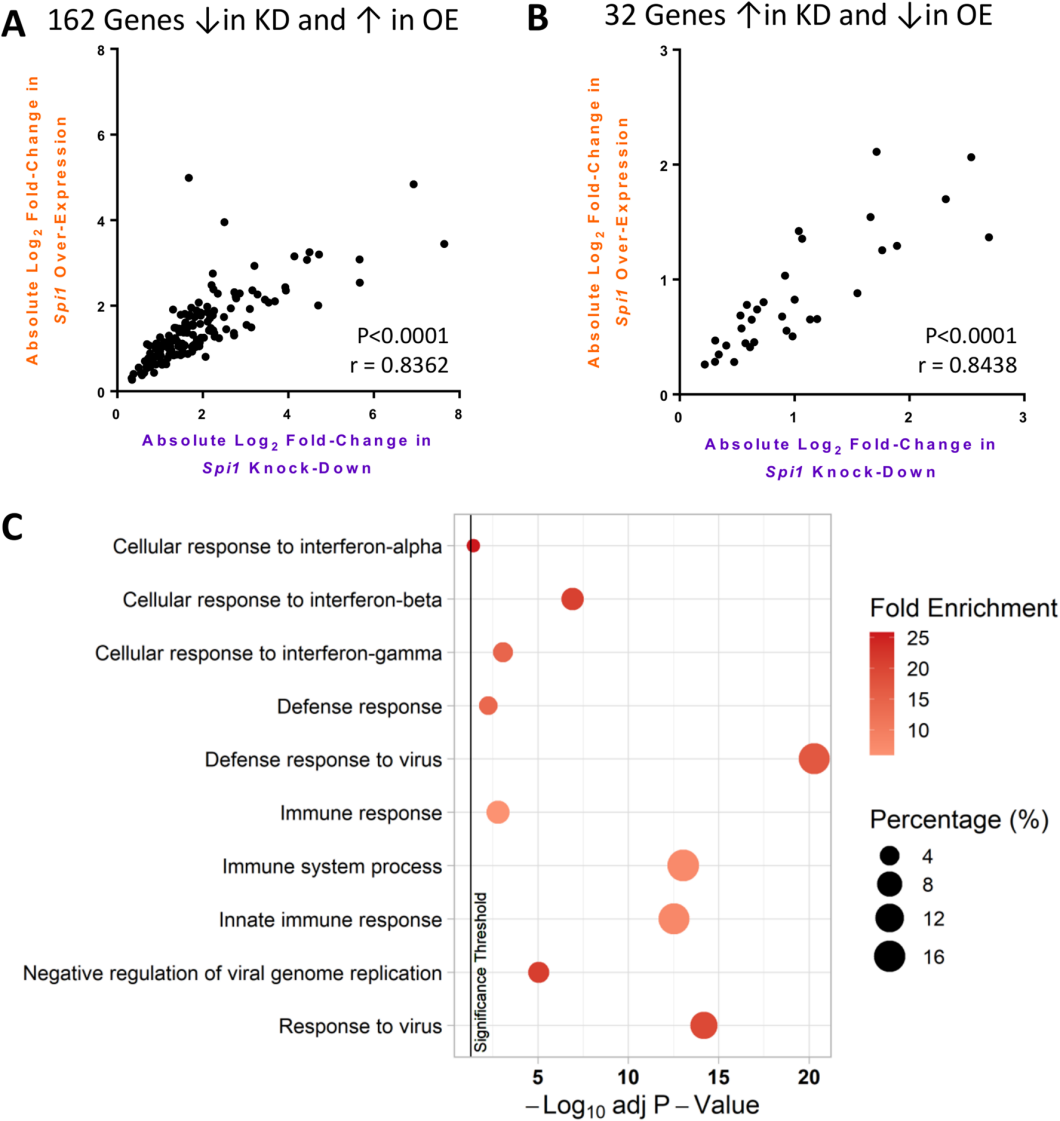
Absolute Log_2_ fold-changes in *Spi1* dose-sensitive genes. (**A**) Absolute log_2_ fold-change values were used the 162 genes where *p* ≤ 0.05 with negative fold-change values in the *Spi1* knock-down dataset (purple) and positive fold-change values in the *Spi1* over-expression dataset (orange) were assessed separately from the 32 genes where *p* ≤ 0.05 and *Spi1* dose had the opposite effect on expression (**B**). Two-tailed Spearman Rank test identified significant correlations between the log_2_ fold-changes in both graphs (approximate *p* < 0.0001 and *r* > 0.8). (**C**) Top 10 most significantly altered “Biological Processes” according to the Benjamini–Hochberg adjusted *p* value. The bubble size indicates the percentage of the gene list aligned to this pathway. The colour indicates the fold enrichment, which is the proportion of genes present in this list compared to the background gene expression. The vertical black line indicates the *p* value threshold of 0.05. Figures (**A**, **B**) were made in GraphPad PRISM 6 (version 3.07) and (**C**) using the ‘tidyverse’^37^ and ‘ggpubr’^38^ packages in R^39,40^.

The absolute log_2_ fold-changes of 162 genes reduced by *Spi1* knock-down and increased by *Spi1* over-expression significantly correlated to each other (Fig. 3A) and the 32 genes that were increased by *Spi1* knock-down and reduced by *Spi1* over-expression (Fig. 3B) were also found to correlate (Two-tailed Spearman Rank Test approximate *p* < 0.0001 and *r* > 0.8 for both). This further supports the hypothesis that the expression of these genes is controlled in a *Spi1* dose-sensitive manner. Collective analysis of the 194 *Spi1* dose-sensitive gene list using GO terms through DAVID indicated an enrichment of immune response genes (Fig. 3C), particularly pathways linked to interferon and viral defence responses. These results suggest that a high microglial *Spi1* expression may result in more responsive microglia that produce more interferon, though further work would be required to confirm this experimentally.

### Several gene clusters implicate immune system responses

**Figure 4:**
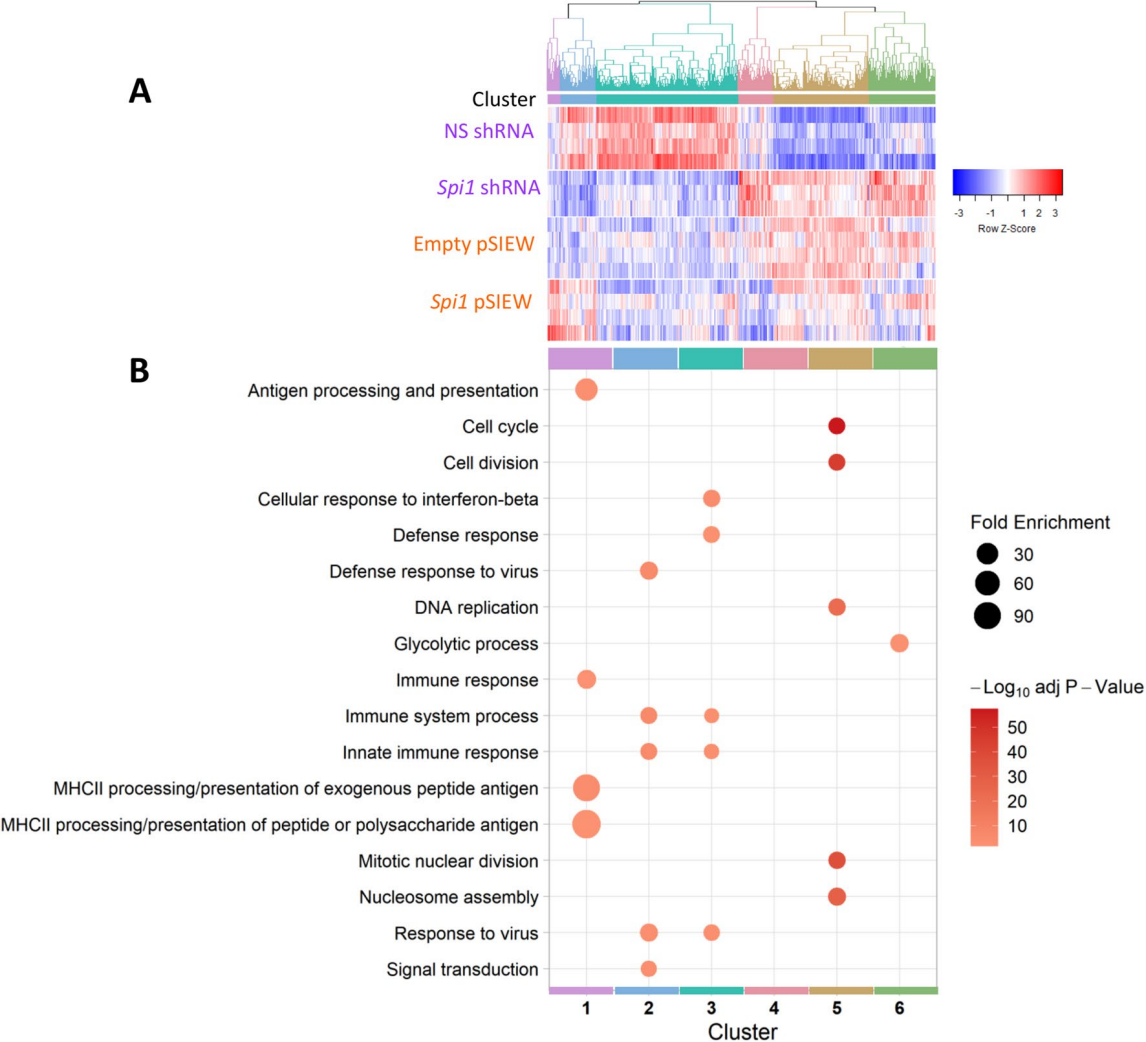
Hierarchical clustering analyses with the 20 most siginificantly changed biological pathways each cluster assessed using DAVID. (**A**) Hierarchical clustering of rows, where each row represents the scaled log_10_ FPKM values for each gene, with dendrogram higlighting cluster boundaries. The Pearson correlation was used to calculate the Z-Scores and UPGMA agglomeration methods were used. These 6 clusters were produced using the cutree function at a height of 1.59. (**B**) The gene lists were assessed using DAVID and a bubble plot created to highlight the top 5 most significatly enriched pathways (*p* value threshold of < 0.05) in each gene cluster. Cluster 1 contains genes that had increased expression following a higher *Spi1* expression and are mainly linked to immune responses and MHCII antigen presentation. Cluster 2 appears to contain more of the *Spi1* dose-sensitive genes and are enriched for viral/immune response signalling pathways. Genes in Cluster 3 had a lower expression in the *Spi1* shRNA dataset and are related to immune response and interferon signalling pathways. Cluster 4 contained no significantly enriched pathways using the *p* ≤ 0.05 threshold. Cluster 5 contains multiple genes related to cell cycle and DNA replication pathways, whose expression was increased in the *Spi1* shRNA samples. The bubble size denotes the fold enrichment and the colour Benjamini–Hochberg adjusted *p* values. Figures were made with the ‘tidyverse’^37^, ‘gplots’^42^, ‘plotly’^43^, ‘dendextend’^44^ and ‘colourspace’^45^ packages in RStudio^39,40^, code adapted from^46,47^ complete Markdown is available in Supplementary Information.

The data were assessed using hierarchical clustering of the FPKM expression values (Fig. 4). Six discrete clusters were identified, and gene lists were assessed using DAVID as before. Genes with an increased expression in microglia over-expressing *Spi1* (Cluster 1) are enriched for pathways involving MHCII processing and presentation of antigens. The genes in Cluster 2 appear to be the most *Spi1* sensitive, where expression was reduced in the *Spi1* shRNA and increased in the *Spi1* over-expression samples compared to relative controls. Most pathways associated with the genes in this cluster are related to the immune response including the response to viruses. Cluster 3 contained genes that were down-regulated in the *Spi1* shRNA samples compared to the controls and were related to interferon signalling and immune response pathways. Cluster 5 contains genes that had a higher expression in the *Spi1* shRNA samples compared to the control shRNA samples. The GO TERMS associated with the genes in this cluster include those related to cell cycle and DNA replication pathways. Overall, this reinforces previous reports that reduced *Spi1* seems to impact cell cycle which was not unexpected given the involvement of PU.1 in survival signalling^11,41^. A lowering of *Spi1* appeared to reduce activation of immune response related pathways while increasing *Spi1* expression seemed to have the opposite effect.

### PU.1 dose-related transcriptomic differences were not limited by direct binding

**Figure 5:**
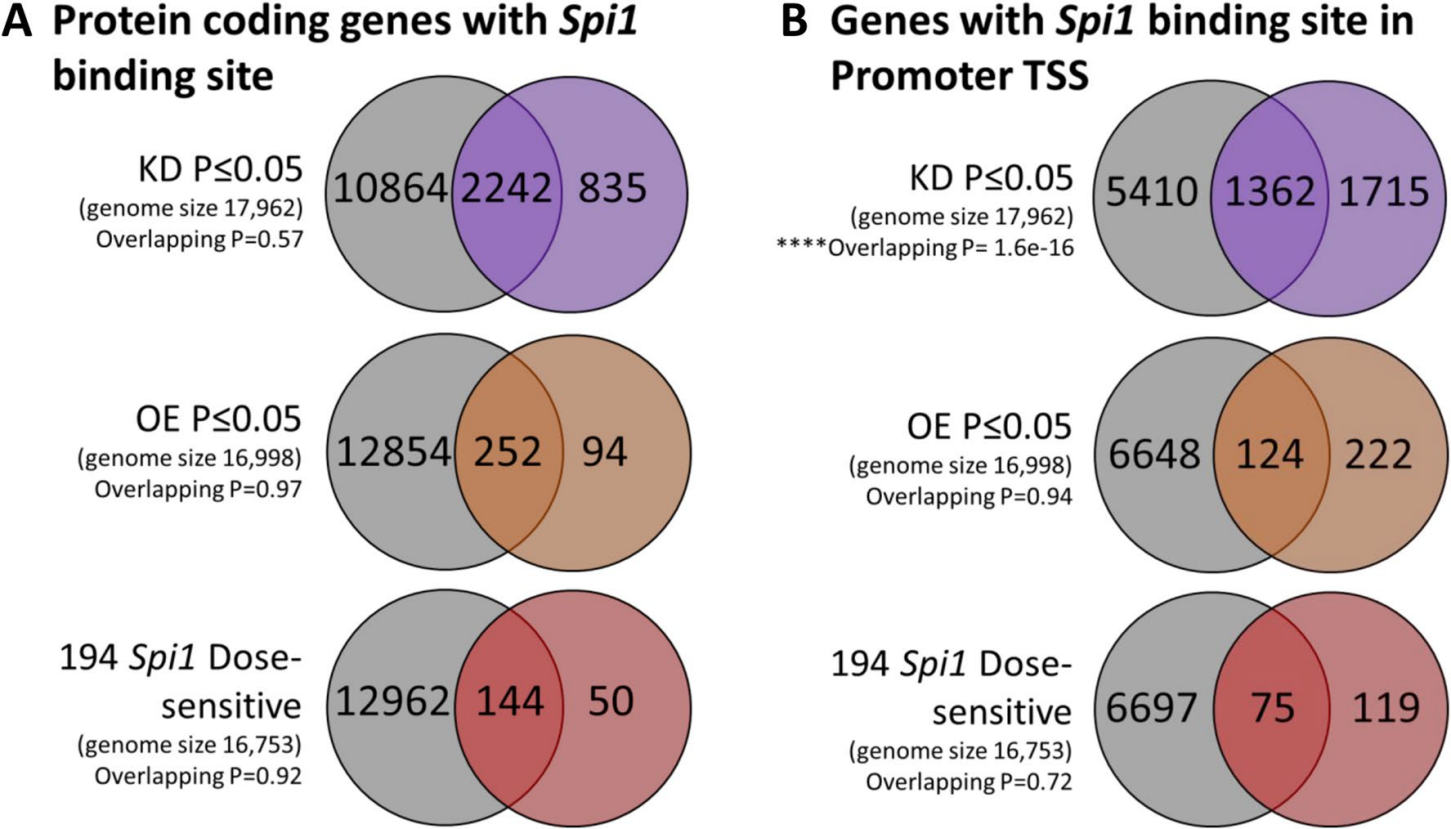
Comparison of *Spi1* KD, *Spi1* OE and 194 *Spi1* Dose-sensitive genes to *Spi1*/PU.1 Chip-Seq dataset. *Spi1* binding sites were determined from PU.1 Chip-Seq published data available online^30^ as described in methods. Fisher’s exact tests were used to determine if there was a significant overlap between the datasets (**A**) includes the results from all protein coding genes with a *Spi1* binding region (defined by Chip-seq in grey) that are also expressed in KD (purple), OE (orange) RNA-seq datasets or the *Spi1* dose-sensitive genes (red). (**B**) The proportion of genes expressed in the RNA-seq datasets that contained a *Spi1* binding site within the promoter region. The KD dataset contained a significant number of genes with a *Spi1* binding sequence in the promoter (Fisher’s exact test, overlapping *p* value = 1.6 × 10^−16^). No significant enrichment was noted in either the *Spi1* OE or dose sensitive genes. Figures were made in PowerPoint based on results from ‘GeneOverlap’^48^ package in RStudio^39,40^.

Changes in gene expression after manipulation of PU.1 could arise from changes in direct DNA binding or via secondary effects. To identify possible direct targets of PU.1 that were differentially expressed, the proportion of protein coding genes that contained a PU.1 binding site sequence, especially within the promoter region, were assessed utilising a published ChIP-Sequencing (ChIP-Seq) dataset available online^30^. Figure 5 shows ~ 70% of genes differentially expressed in the *Spi1* RNA-Seq datasets contained a PU.1 binding site and therefore expression was potentially directly modified by *Spi1*. Moreover, 30–40% of genes in *Spi1* KD and *Spi1* OE datasets had PU.1 binding sites within the promoter region suggesting their expression was directly altered by *Spi1*/PU.1. These proportions remained consistent even in the *Spi1* dose-sensitive subset. Only differentially expressed genes in the *Spi1* KD were enriched for PU.1 binding sites in the promoter region (Fisher’s exact test, *p* value of overlap = 1.6 × 10^−16^). Given the proportion of overlapping genes was similar across all three test sets (*Spi1* KD, *Spi1* OE and *Spi1* dose-sensitive) it seems *Spi1* was not limited to directly regulating genes, but likely indirectly mediates expression of multiple other genes via secondary downstream processes.

### Comparison to human AD risk genes

**Figure 6:**
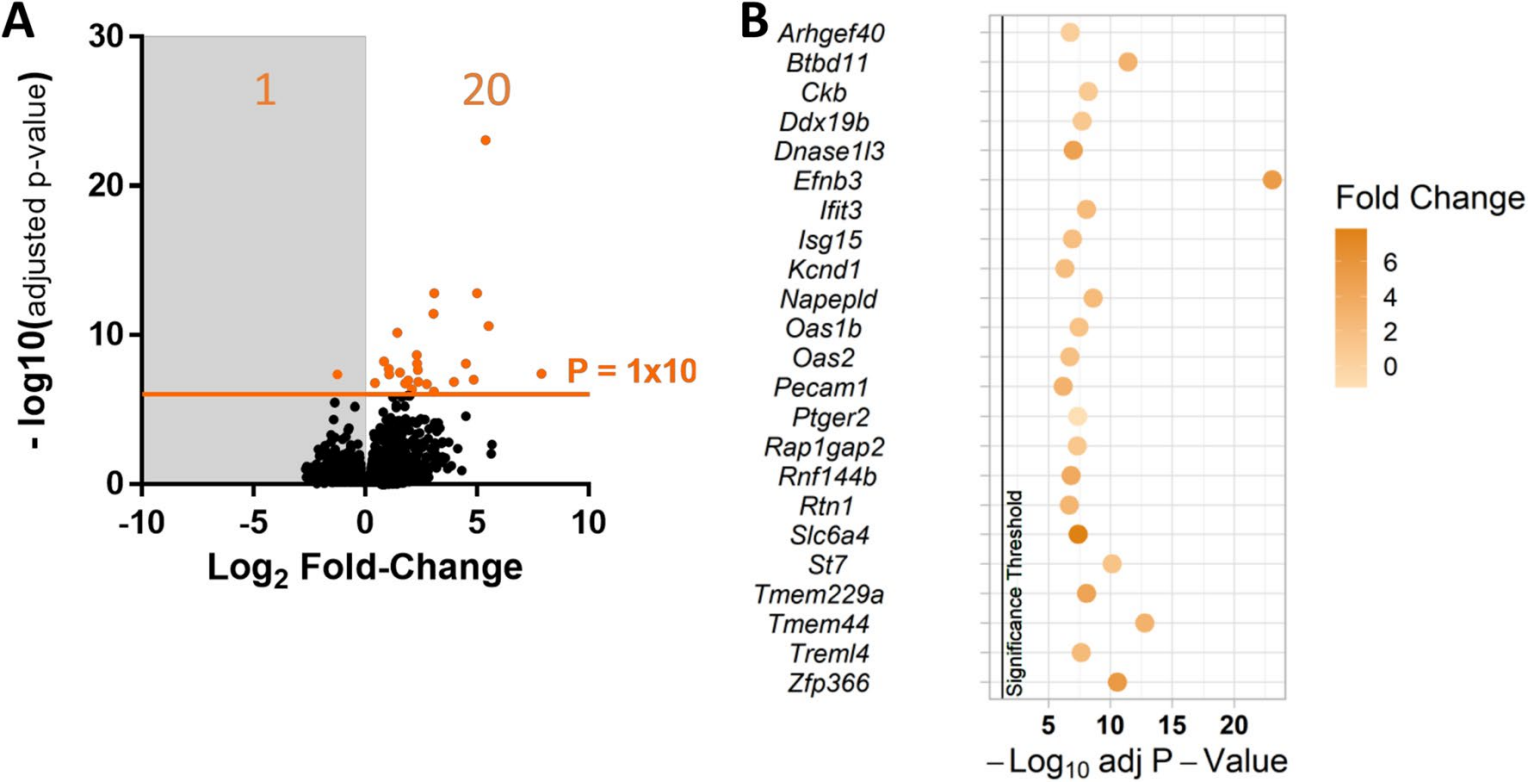
Summary of *Spi1* over-expression genes that were significantly enriched in the IGAP dataset. (**A**) Volcano plot of the *Spi1* over-expression gene expression changes relative to the empty vector control, highlighting the *p* ≤ 1 × 10^−6^ threshold that was shown to be significantly enriched by MAGMA analysis in the IGAP dataset (MAGMA’s empirical multiple testing corrected *p* value 0.035). The adjusted *p* value for this gene set was 0.0033 and an enrichment effect size of 0.51. (**B**) Bubble plot of the genes from the *Spi1* over expression dataset that were enriched for AD genetic risk via MAGMA comparison to the human IGAP database, against the Benjamini–Hochberg adjusted *p* values from the *Spi1* over-expression RNA-sequencing data with the fold-change shown in colour. The vertical black line indicates the *p* value threshold of 0.05. Figure (**A**) was made in GraphPad PRISM 6 (version 3.07) and (**B**) using the ‘tidyverse’^37^ and ‘ggpubr’^38^ packages in in RStudio^39,40^.

Protein network analysis suggest *Spi1*/PU.1 is one of several “hub” genes within a network of AD risk genes^5^, which was further supported by Cis-eQTL analyses in monocytes and macrophages^7^. The RNA-Sequencing profiles generated in this paper were compared to the International Genomics of Alzheimer’s Project (IGAP) dataset, to investigate if there was an enrichment of AD genetic risk within the differential expressed sets of genes. Supplementary Table 2 denotes the gene sets used in this MAGMA analysis. In the *Spi1* over-expression dataset a 21 gene set (Fig. 6), corresponding to a Benjamini–Hochberg adjusted *p* value of ≤ 1 × 10^−6^ for differential expression, were enriched for AD genetic risk (adjusted *p* value = 0.035). The individual IGAP *p* values can be viewed alongside the corresponding *Spi1* over-expression data in Supplementary Table 3. This gene list (Fig. 6B) contained *Ifit3, Oas1b* and *Oas2* which GO analysis linked to the interferon response pathway and *Rnf144b* and *Treml4* which are related to antigen presentation pathways, implicating the immune system response in AD pathology.

## Discussion

Manipulation of *Spi1* expression in primary mixed glia cultures were used to identify how microglial gene expression was altered following modest changes to *Spi1*/PU.1 (Fig. 1). Analysis of the RNA-sequencing datasets produced from these cultures have provided insight into how *Spi1* dose affects the microglia transcriptome. Briefly *Spi1* knock-down resulted in changes to gene expression of components of cell cycle checkpoint pathways while over-expression of *Spi1* altered enriched pathways related to MHCII, interferon response and viral response (Figs. 3, 4). Comparing these RNA-Seq datasets to ChiP-Seq data suggests that approximately 70% of the differentially expressed genes were likely directly altered by *Spi1* binding whereas the rest appeared to be altered via other mechanisms (Fig. 5).

Strengths of this experimental approach include assessing the bidirectional impact of *Spi1*/PU.1 changes (Fig. 1) on the microglial transcriptome in an unbiased manner in primary cell cultures. We supplemented our cultures with TGF-β which partially compensates for the loss of complex environmental cues microglia would normally receive. However, it does not fully replicate the in vivo context and there were no suitable transgenic mice available to study the impact of *Spi1* dose on microglia in situ. The reduction in *Spi1* FPKM values and PU.1 protein levels can be clearly seen in both microglia (Fig. 1D and Supplementary Fig. 3B) and in RAW264.7 cells (Supplementary Fig. 2). Figure 1E clearly shows that the *Spi1* pSIEW plasmid results in increased *Spi1* mRNA and PU.1 expression in microglia.

PU.1 has been previously linked to proliferation in macrophages. Bone-marrow derived Macrophages over-expressing PU.1 increased GM-CSF and M-CSF dependent proliferation and cell number, the opposite was seen in cells transfected with an anti-sense PU.1^9^. However, there are some conflicting reports in the literature as to the role of PU.1 in microglia proliferation. In this model absolute cell numbers did not differ between *Spi1* shRNA and control shRNA infected microglia, neither were there significant fold-change differences between normalised cell cycle data (Supplementary Fig. 3). In human microglia cultures an siRNA mediated reduction of PU.1 resulted in a lower cell number, disintegration and rounding of some cells but at the final timepoint viability appeared unaffected. This suggests some microglia could survive with reduced PU.1^41^. Another similar study observed no reduction in microglia number, though PU.1 loss was measured at a culture level so the reduction could not be quantified on a single-cell level^31^. In summary, we did not observe significant changes in the proportion of cells in each phase of the cell cycle following a reduction in PU.1 (Supplementary Fig. 3), despite the differential expression of cell cycle associated genes within the *Spi1* KD RNA-Seq dataset (Fig. 2).

PU.1 has been shown to bind the promoter of the Csf1-receptor (Csf1r) increasing expression of Csf1-receptor^49^, which is highly linked to microglia survival and proliferation. In BV-2 cells a reduction in PU.1 also results in a reduced *Csf1r* expression^7^ though no cell number/viability data was provided. It was recently shown that BV-2 cells with diminished PU.1 expression were more vulnerable to caspase-dependent cell death, while PU.1 over-expressing cells appear to have delayed onset of death. Baseline cell viability did not appear to be impacted by PU.1 modulation in these cells^32^. In the *Spi1* RNA-seq datasets generated in this paper *Csf1r* expression was not significantly altered (Benjamini–Hochberg adjusted *p* values 0.665 in KD and 0.999 in OE), though the impact on Csf1r protein expression was not investigated here. Given that Csf1r inhibitors have been shown to prevent AD-related microgliosis and partially ameliorate disease pathology in vivo^33–36^ it would be interesting to see if Csf1R protein was affected in *Spi1* shRNA infected microglia and if the reduction in PU.1 had a similar impact to Csf1r inhibitors on disease progress.

Over-expression of *Spi1* resulted in differentially expressed genes enriched for MHCII related pathways, interferon response pathways and response to virus pathways (Fig. 2B). Though there may be minor concerns that the significance of viral immune response pathways could be an unintended effect of using lentiviruses to manipulate *Spi1* expression this is unlikely as comparisons were made relative to an almost identical control virus that lacked the *Spi1* coding sequence. Additionally, this study identified a subset of 194 genes that appear to be *Spi1* dose sensitive, linked to GO terms such as “Cellular response to interferon” and “Defence response to virus” (Fig. 3). Therefore, an increased level of *Spi1* appears to cause transcription of genes associated with an inflammatory phenotype that might be relevant to AD genetic risk mechanisms (Fig. 6).

Previous work has identified that *Spi1* is able to influence multiple gene networks in microglia^5,7,31^ and has been proposed as central to a network of AD risk genes that are conserved between species^50^. Basal cytokine expression was higher in BV-2 cells over-expressing PU.1, and was further potentiated by LPS stimulation compared to control samples^32^. Media taken from LPS treated PU.1 over-expressing BV-2 cells promoted a reactive astrocyte phenotype which was not observed in astrocytes treated with media from LPS treated BV-2 cells with reduced PU.1 expression^32^. Together these results suggest that PU.1 dose likely modulates the cytokine response in stimulated BV-2 microglia cells. In co-transfected NIH-3T3 fibroblasts PU.1 was required to bind either IFN Regulator Factor 4 (IRF-4) or IFN Consensus Binding Protein (ICSBP) for maximal induction of an IL-1β reporter assay^51^. Therefore, it is likely that increased *Spi1*/PU.1 expression levels in microglia may result in a more inflammatory microglial profile, potentially in conjunction with additional transcription factors. GO analysis of the *Spi1* knock-down and over-expression datasets suggest that the level of *Spi1* may impact microglial phenotype. Work in BV-2 cells suggests that reduced PU.1 results in a less reactive phenotype whereas PU.1 over expression primes cells for a more reactive response^32^. Further profiling is needed to ensure the pathway analysis matches the phenotype presented in this model or ideally in vivo.

Several gene-expression studies in AD model mice have highlighted *Spi1* as a target of interest. Salih et al. identified a network of microglial genes expressed by amyloid-associated microglia, collated from five transgenic mouse lines, including *Spi1*, and 74 other genes that were identified as *Spi1* dose-sensitive in this study^52^. These genes included *Oas1b*, *Oas2*, *Ifit3* which were all highly differentially expressed in the *Spi1* over-expression dataset (Fig. 6) and are linked to interferon signalling^53^. Sierskma et al*.* identified 18 genes that both overlapped with AD GWAS datasets and were maintained between young and old APP transgenic mice, which were predominantly expressed by microglia and appear to be regulated by *Spi1*^50^.

There are several reports in the literature that imply human and mouse microglia have phenotypic differences. For example, work in primary human microglia suggests that the responses to IFN-γ, TGF-β1 and M-CSF may be species specific^54^. As this work was carried out in primary mouse cultures there could be concerns that the impact of reduced or increased *Spi1*/PU.1 might not be present in human biology.

However, studies investigating PU.1 in human microglia suggest there are shared *Spi1*/PU.1 dose dependent pathways in microglia. In addition to *SPI1* binding sites being located near other AD risk genes or regulatory elements^5,7,8^, *SPI1* risk alleles associated with a higher gene expression have been shown to lower the age of onset in AD^7^. Transcriptional profiles of microglia isolated from frozen human AD and control cortical tissue suggest an overall species difference in the disease associated signature, though an increased *SPI1* expression was measured in AD samples compared to controls^55^ validating previous observations by others^31^.

In primary human microglia cultures siRNA mediated reductions in PU.1 resulted in a decrease in Amyloid-β 1-42 peptide phagocytosis^41^, in a similar manner to observations in BV-2 cells^7,32^. Analysis of mixed microglia/pericyte cultures where PU.1 was reduced via siRNA showed reduced expression of DAP12 and HLA-DR/DP/DQ^31^. Moreover, similar gene expression changes to immune response and lipid metabolism genes were observed in BV-2 cells and human iPSC microglia following PU.1 siRNA knock-down^32^. Together these studies show that while the impact of *SPI1* dose on microglia phenotype needs to be assessed in both species, some functional changes appear common to both human and mouse microglia.

In summary, disease relevant reductions in *Spi1* highlighted dysregulation of genes involved in cell cycle checkpoint pathways. Modest increases in microglial *Spi1* expression results in dysregulation of genes linked to immune response and interferon signalling, suggestive of a more pro-inflammatory microglial phenotype. This study highlights how relatively modest changes to *Spi1*/PU.1 expression alone can have a large impact on the microglial transcriptome of primary mixed-glial cultures, providing candidate pathways for future studies investigating *Spi1* dependent processes and AD relevant biology.

## Methods

### Primary mixed glia cultures

Brains from 8-week-old C57BL/6 J mice (Charles River) were collected and transported in Hank’s Balanced Salt Solution (HBSS without Mg^2+^ or Ca^2+^; Gibco) on ice. These culture experiments were repeated 10 times (n = 10), 4 were used to generate RNA-Sequencing replicates and 6 for subsequent validation experiments. For most cultures three brains were sufficient per experiment (one 6 well-plate), though for some experiments more cells were required so cultures were scaled up accordingly. Brains were digested using the Neural Tissue Digest Kit-P in C-tubes as per manufactures directions (Miltenyi Biotec). Two brains were digested in each C-tube using program 37_ABDK on the GentleMACS OctoDissociator (Miltenyi Biotec) to produce a single cell suspension. Cell suspensions were passed through a 70 μM strainer (Falcon) into a 50 mL tube and centrifuged at 300 × *g* for 7 min. The supernatant was aspirated and the pellet resuspended in 10 mL Dulbecco’s Minimum Essential Media (DMEM containing 4.5 g/L D-Glucose, GlutaMAX and supplemented with 15% (v/v) heat-inactivated Foetal Bovine Serum (FBS) and 100 units/mL (v/v) Penicillin and 100 μg/mL Streptomycin (v/v); all Gibco) before centrifugation at 300 × *g* for 7 min. The supernatant was aspirated, and the pellet resuspended in 2 mL per brain of DMEM media containing 10 ng/mL recombinant murine M-CSF (Peprotech). Cell suspensions were then pooled as required and placed in a 6-well plate (2 mL per well) or 100 mm × 20 mm (diameter × height) tissue culture plates, 12 mL per plate, and moved to a humid incubator with 95% air and 5% CO_2_ overnight to allow the microglia to adhere.

Myelin was resuspended in the media (with 24 h of digest) by gentle plate agitation and the media containing the myelin debris was discarded. The cell monolayer was carefully washed with DMEM media. The wash media was then replaced with fresh DMEM media with 10 ng/mL M-CSF and this was again replaced two days later. On day 5 of culture the media was replaced with DMEM media containing 10 ng/mL M-CSF and 50 ng/mL recombinant mouse TGF-β1 (eBioscience or Biolegend). TGF-β supplementation was used to promote a more homeostatic phenotype in vitro^56,57^. Recombinant murine TGF-β1 was purchased either from eBioscience or Biolegend, both of which were produced in a similar way. The Biolegend recombinant TGF-β1 was diluted to a 100 μg/mL stock in an equal volume of sterile filtered 2% BSA, 0.2 M glycine in DPBS to match the eBioscience product.

From this point forward all culture media contained M-CSF and TGF-β1 was replaced every two days. On day 10, these cultures were infected with *Spi1* shRNA, control shRNA, *Spi1* pSIEW or pSIEW lentivirus particles as appropriate. To achieve this the culture media was replaced with 3 mL fresh media and 100–300 μL of lentivirus was added to each plate. The volumes of *Spi1* targeting and control viruses were kept the same within each experiment. After 6 h each plate received an additional 3 mL of culture media. The media on these cultures continued to be changed every 2 days, supplemented with M-CSF and TGF-β1 as before.

All animal experiments were conducted in accordance with UK Home Office Guidelines and Animal [Scientific Procedures] Act 1986 which included full review and approval by the local ethical review board (Animal Welfare and Ethical Review Body, AWERB, of the Biological Standards Committee) and the granting of a UK Home Office Project Licence. The study was conducted in compliance with the ARRIVE guidelines.

### Sample processing for RNA-sequencing

After 21 days the media was removed, set-aside, and the mixed glial cells harvested by incubating with ~ 10 mL Accumax (Sigma) at 37 °C for 10–20 min. The monolayer was gently washed with DMEM media and any remaining attached cells were carefully removed using a plastic scraper (Greiner). The cell suspension was added back to the media and centrifuged at 300 × *g* for 7 min. The supernatant was removed via pipetting and the cell pellet was resuspended in 0.5% BSA in DPBS with a 1:1000 dilution of LIVE/DEAD near-IR staining solution for 30 min on ice per manufacturers direction (Molecular Probes).

Samples were centrifuged at 300 × g for 7 min and the supernatant aspirated via pipette. Each sample was resuspended in 500 µL block solution (4 μg/mL Rat anti-mouse FcγRII/III (clone 2.4G2) and 5% (v/v) filtered rabbit serum in 0.5% BSA (w/v), 5 mM EDTA in DPBS) and kept on ice for 10 min. CD11b (Clone M1/70 PerCP-Cy5.5 conjugate, BD Biosciences final dilution 2 μg/mL) and CD45 (Clone 30-F11 eFlour 450 conjugate, eBioscience final dilution 2 μg/mL) antibodies were diluted to a 4 μg/mL concentration in 0.5% BSA (w/v), 5 mM EDTA in DPBS. 500 μL of the antibody staining solution was added to each sample, to give a final antibody concentration of 2 μg/mL, and the samples were incubated on ice for a further 15 min. The tubes were then centrifuged at 300 × *g* for 7 min and the supernatant removed.

The cells were resuspended in 1 mL of 0.5% BSA (w/v), 5 mM EDTA in DPBS and kept cool before undergoing fluorescent activated cell sorting on a FACS Aria III (BD Biosciences). Dead cells were excluded from the sort and microglia were selected using CD11b/CD45 double staining and GFP as a marker of infection. The sorted cells were then pelleted via centrifugation, the supernatant aspirated and the pellet lysed for RNA using the Mini RNeasy kit (Qiagen) per manufacturer’s directions.

### Lentivirus preparation

The shRNA vector construct and lentivirus production method has been described in^58^ and sucrose-gradient purification in^59^. The insert sequences for the control shRNA and *Spi1* shRNA can be seen in Table 1.

**Table 1:** shRNA sequences including termination sequence (bold).

***Spi1* shRNA sequence (5′ → 3′)**
GATGTGCTTCCCTTATCAAACCTCGAGGTTTGATAAGGGAAGCACATC**TTTTT**
**Control shRNA sequence (5′ → 3′)**
GTCTCGCTTGGGCGAGAGTAAGTAGTGAAGCCACAGATGTACTTACTCTCGCCCAAGCGAGAC**TTTTT**

The over-expression plasmid used a spleen focus forming virus (SFFV) promoter to drive expression of murine *Spi1* (Ensemble reference CCDS 16,425.1). An Internal Ribosome Entry Site (IRES) was used to initiate translation of a downstream eGFP reporter. Lentiviruses were purified by overlaying the virus containing media over a 20% sucrose solution before ultracentrifugation at 26,000 rpm for 90 min at 4 °C in a SW28Ti swinging-bucket ultracentrifuge rotor assembly in an Optima XPN-80 Ultracentrifuge (Beckman Optima Ultracentrifuge) and the viral pellet was resuspended in AimV media.

### RNA-sequencing data analysis

RNA was isolated from cell sorted GFP + microglia using the RNeasy Mini kit (Qiagen) per manufacturer’s instructions and eluted in nuclease free water. RNA integrity and concentration were assessed using the Agilent 2100 Bioanalyzer (Aligent). Complementary (cDNA) libraries were generated using the Truseq stranded total RNA with Ribo-Zero GOLD kit (Illumina). Paired end sequencing was then performed using the Illumina HiSeq 2500 sequencing platform to a read depth of between 30 and 40 million pairs.

The FASTQ files were processed with Trimmomatic^60^ to remove paired-end reads and quality was confirmed in FastQC using default parameters^61^. Following this sequence reads were mapped to the mm10 genome (GRCm38) using the STAR pipeline^62^ and featureCounts was used to assign counts to transcripts^63^ with the GRCm38.84 Ensembl gene build GTF. The Ensembl FTP site was used to download the reference genome and GTF^64^. Differential gene expression was performed using the DESeq2 package^65^. Genes that were not significant after the differential expression analysis were discarded; significance was defined as an adjusted *p* value (Padj) of < 0.05 (Benjamini–Hochberg correction for multiple testing).

#### Assessing PU.1 promoter binding sites

A microglial PU.1 ChIP-sequencing (ChIP-seq) dataset (GSM1533906) isolated from C57BL/6 mice of a similar age (8–9 weeks)^30^ was accessed via the Cistrome database^66^. This dataset was then run through HOMER^28^ to generate an annotated peak file with genomic features. The promoter region was defined as 1000 base pairs upstream or 100 base pairs downstream of the transcription start site.

Duplicates were removed from the annotated ChIP-seq dataset and compared to gene discoveries from knock-down, over-expression and dose-sensitive *Spi1* RNA-sequencing datasets. The background genome size was the number of genes detected in each RNA-Sequencing dataset defined as either all control and/or all experimental samples had a raw read count of ≥ 5. In the *Spi1* knock-down dataset 17,962 genes were detected and 16,998 genes in the *Spi1* over-expression data. For the 194 *Spi1* dose-sensitive genes a merged list of genes expressed in either the *Spi1* knock-down or over-expression RNA-sequencing datasets was generated, and duplicates removed, to give a background gene number of 16,753.

Finally, the ChIP-seq datasets were compared against their respective RNA-Seq dataset (Padj ≤ 0.05) in R using the ‘tidyverse’ and ‘GeneOverlap’ packages. Here, a Fisher’s exact test was utilised to confirm if there was any significant enrichment of PU.1 ChIP-seq binding sites in genes expressed in *Spi1* knock-down, over-expression or dose-sensitive datasets.

#### Gene ontology analysis

Enriched gene Ontology (GO) terms were identified using the Database for Annotation, Visualization and Integrated Discovery (DAVID, version 6.8) and compared against a background of genes expressed in at least one *Spi1* RNA-seq dataset (16,998 genes). Gene expression in the *Spi1* RNA-seq datasets was defined as having a raw read count ≥ 5 in all control and/or all experimental samples. The most significantly altered terms from the GOTERM_BP_DIRECT list, henceforth called Biological Process GO Terms, were downloaded for subsequent analysis. Bubble plots were generated in R using the ‘ggplot2’ package, and an adjusted *p* value cut-off of 0.05 as indicated on each plot. Data utilised in these plots included the Benjamini–Hochberg adjusted *p* value, the percentage of the genes from the RNA-seq datasets that aligned to this pathway and the fold enrichment, which was defined as the proportion of genes present in this list compared to the *Spi1* RNA-Seq gene expression background.

#### Hierarchical clustering analysis

Hierarchical clustering was performed utilising ‘gplots’, ‘dendextend’ and ‘colorspace’ packages in R with code adapted from^46,47^, to produce a heatmap and dendrogram to better visualise the clusters. Complete markdown can be found in Supplementary Methods. FPKM values were log_10_ transformed, and z-scored (Pearson Correlation method) prior to clustering. Hierarchical clustering resulted in six distinct clusters which are highlighted in different colours in the dendrogram (Fig. 4).

#### Enrichment of association signal in IGAP GWAS data

As *Spi1* has been implicated in the regulation of other AD risk gene expression, both RNA-Seq datasets were tested for enrichment of association with AD risk in the International Genomics of Alzheimer’s Project (IGAP) GWAS dataset^67^. Firstly, the BioMart feature in Ensembl was used to convert mouse genes differentially expressed in the *Spi1* knock-down and over-expression datasets into human orthologs^68^.

Gene sets were then determined for each *Spi1* RNA-Seq dataset using *p* value cut-offs between 0.05 and 1 × 10^−10^. Direction of differential expression was not considered when defining gene sets. These gene sets (Supplementary Table 2) were tested for enrichment in the IGAP dataset using Multi-marker Analysis of GenoMic Annotation (MAGMA) analysis^69^.

### Flow cytometric analysis of PU.1

Mixed glial cultures were harvested on day 21. Culture media was carefully removed and retained. Cells were detached by incubating with Accumax for 10–20 min at 37 °C, added to the appropriate culture media and centrifuged at 300 × *g* for 7 min. Approximately 2 × 10^5^ cells per sample were fixed in 4% formalin solution (in DPBS) on ice for 30 min. Formalin was removed by centrifugation (300 × *g* for 7 min) and cells were permeabilised on ice in 90% ice cold methanol (v/v in PBS). Samples were centrifuged at 300 × *g* for 7 min and the supernatant discarded. To ensure the methanol was completely removed an additional wash step using 500 μL of wash solution (0.5% (w/v) BSA, 5 mM EDTA and 2 mM NaN_3_ in DPBS) and centrifugation at 300 × *g* for 7 min. The supernatant was discarded, and the cell pellets were then re-suspended in 50 µL of block solution (5% (v/v) filtered Rabbit Serum, 4 μg/mL Rat Anti-mouse FcγRII/III 2.4G2 clone in wash solution) and incubated on ice for 20–30 min. Following the blocking step 50 μL of CD11b, CD45 and PU.1 antibodies (listed below) were added and cells were incubated for 30–60 min on ice in the dark. The following antibodies were used in Flow cytometric experiments; Anti-CD11b FITC (56C, 8 μg/mL, produced in house), Anti-CD11b BV421 (M1/70, 2 μg/mL, Biolegend), Anti-CD11b PerCP-Cy5.5 (M1/70, 2 μg/mL, BD Biosciences), Anti-CD45 eFlour450 or PE-Cyanine7 conjugates (30-F11, 2 μg/mL, eBioscience), Anti-PU.1 AF647 (7C2C34, 5 μg/mL, Biolegend) and Rat IgG2a,k AF647 Isotype Control (RTK2758, 5 μg/mL, Biolegend).

Cells were washed with wash solution 3 times and centrifuged at 300 × *g* for 7 min. After the final wash the cell pellets were re-suspended in 500 μL of wash solution and acquired on Attune NxT cytometer (Thermofisher). Data was analysed using FlowJo software (version 10; FlowJo LLC). Single colour and isotype controls were used as appropriate. Post data collection relative PU.1 protein levels were determined using the Median Fluorescent Intensity (MFI) values in the PU.1 antibody channel. First the isotype background signal from the same channel was subtracted. These values were then normalised between experiments by diving each value by the average MFI value (minus isotype) of the appropriate control sample.

### Statistics and figures

Statistical analyses were performed using GraphPad PRISM 6 (version 3.07) and GraphPad PRISM 8 (version 8.4.3; both GraphPad Software, Inc.), unless otherwise stated. All statistical tests will be described as appropriate. *p* values of > 0.05 were taken as non-significant (ns). *p* values of ≤ 0.05 will be denoted with a single asterisk*, *p* values of ≤ 0.01 will be written as **, *p* values of ≤ 0.001 by *** and *p* value of ≤ 0.0001 as ****. Figures were made using both GraphPad PRISM 6, GraphPad PRISM 8 (version 3.07 & 8.4.3; GraphPad Software, Inc.) and R Studio (Version 1.2.5042 copyright 2009–2020 RStudio, Inc.^39^) with base R version 4.0.0 (2020–04-24, copyright 2020 The R Foundation for Statistical Computing^40^). The following R packages were used ‘tidyverse’^37^, ‘ggpubr’^38^, ‘gplots’^42^, ‘plotly’^43^, ‘dendextend’^44^, ‘colourspace’^45^ and ‘GeneOverlap’^48^.

## Supplementary Information

Supplementary Information.
