# Supplementary material for: Modest changes in Spi1 dosage reveal the potential for altered microglial function as seen in Alzheimer’s disease
Source: Sci Rep. 2021 Jul 22;11:14935. doi: 10.1038/s41598-021-94324-z (PMC8298495; doi:10.1038/s41598-021-94324-z)
Supplement: Supplementary file 1 — Supplementary Information. [file 41598_2021_94324_MOESM1_ESM.docx]

# Supplementary Figures and Tables

# Modest changes in *Spi1* dosage reveal the potential for altered microglial function as seen in Alzheimer’s disease

Dr Ruth E. Jones^1,2^, Dr Robert Andrews^1,4^, Professor Peter Holmans^3^, Dr Matthew Hill^2^, Professor Philip R. Taylor^1,2,4,*^

*^1^Division of Infection and Immunity, Cardiff University, UK.*

*^2^UK Dementia Research Institute, Cardiff University, UK.*

*^3^Division of Psychological Medicine and Clinical Neurosciences, Cardiff University, UK.*

*^4^Systems Immunity Research Institute, Cardiff University, UK.*

*Address Correspondence to:

Prof. Philip Taylor, UK Dementia Research Institute at Cardiff, Cardiff University, Hadyn Ellis Building, Maindy Road, Cardiff, CF24 4HQ, UK; email: [TaylorPR@cardiff.ac.uk](mailto:TaylorPR@cardiff.ac.uk).


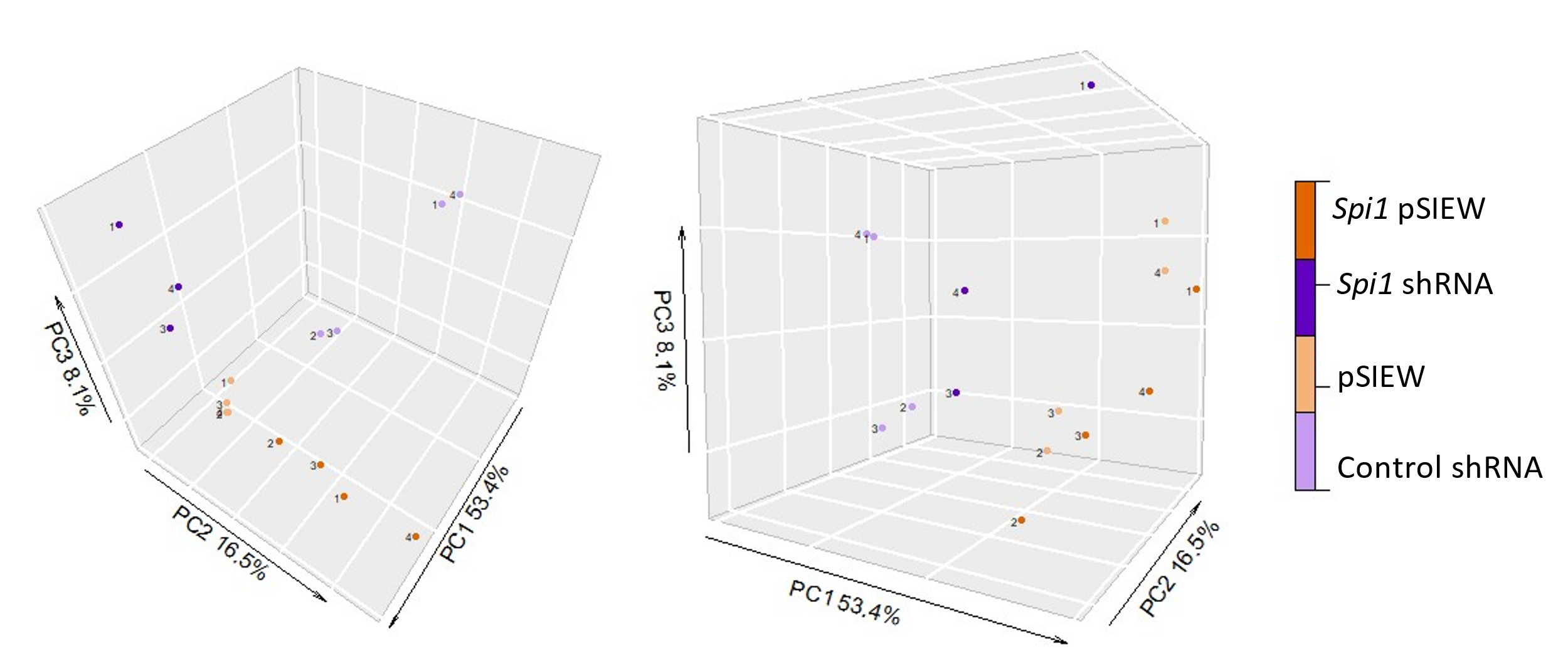


**Supplementary Figure 1- Plot Principal Components 1 and 2-** Each replicate within the sample group seems to have similar eigenvectors to the other samples (n=4 per group, except Spi1 shRNA where n=3). PC1 accounts for approximately half of the variation between samples, and it appears the control shRNA sample group is the most disparate. The top 10 genes that contribute to PC1 linked to processes such as mRNA processing/splicing and metabolic processes. The second and third principal components (PC2 & PC3) appears to represent the impact of Spi1 dose on the microglia transcriptome, as Spi1 pSIEW samples higher than the Spi1 shRNA samples, whereas both controls lie closer to the midline. Figure and analyses were performed using the ‘tidyverse’ [1], ‘corrplot’ [2], ‘factoextra’[3], ‘rgl’ [4]and ‘plot3D’ [5] packages in packages in R [6,7].


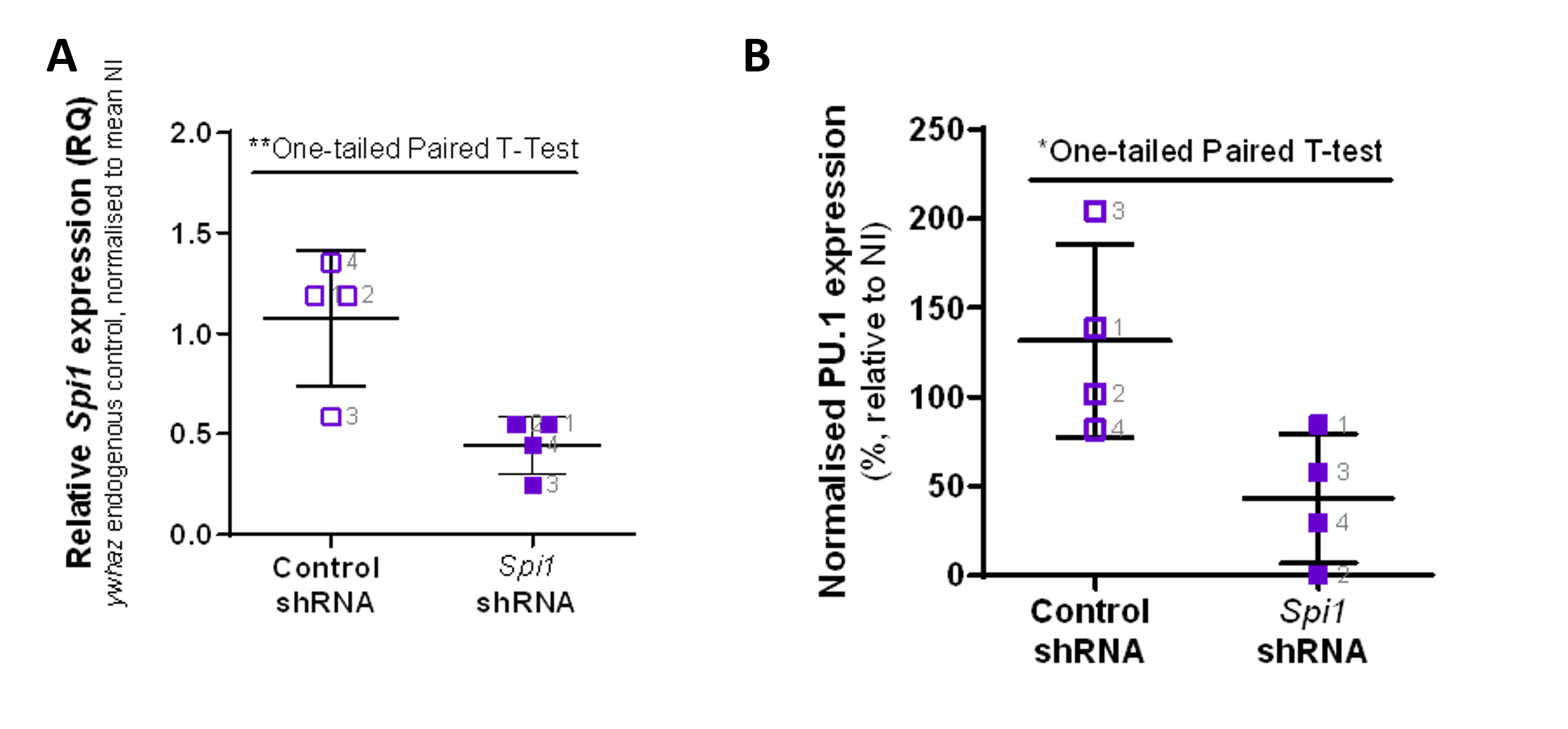


***Supplementary Figure 2- Confirmation of Viral Function.*** *RAW264.7 cells were infected with either Spi1 shRNA or control shRNA lentivirus. After one week cells were harvested and samples divided for PU.1 protein analysis or Spi1 mRNA assessment via qPCR in GFP+ cells.* ***A*** *Spi1 mRNA expression was reduced in Spi1 shRNA infected cells compared to cells infected with control shRNA lentivirus (One-tailed Paired T-test, p-value = 0.0061, n=4).* *Relative gene expression (RQ) was normalised to the mean Spi1 expression of all the non-infected (NI) samples (n=4).* ***B*** *PU.1 protein expression was significantly reduced in cells infected with Spi1 shRNA compared to RAW264.7 cells that were infected with the control shRNA virus (One-tailed Paired T-test, p-value = 0.0145, n=4).* *Individual symbols represented paired experiments (numbered) with error bars representing mean ± SD. Figures were made using GraphPad PRISM 8 (version 8.4.3; both GraphPad Software, Inc.).*


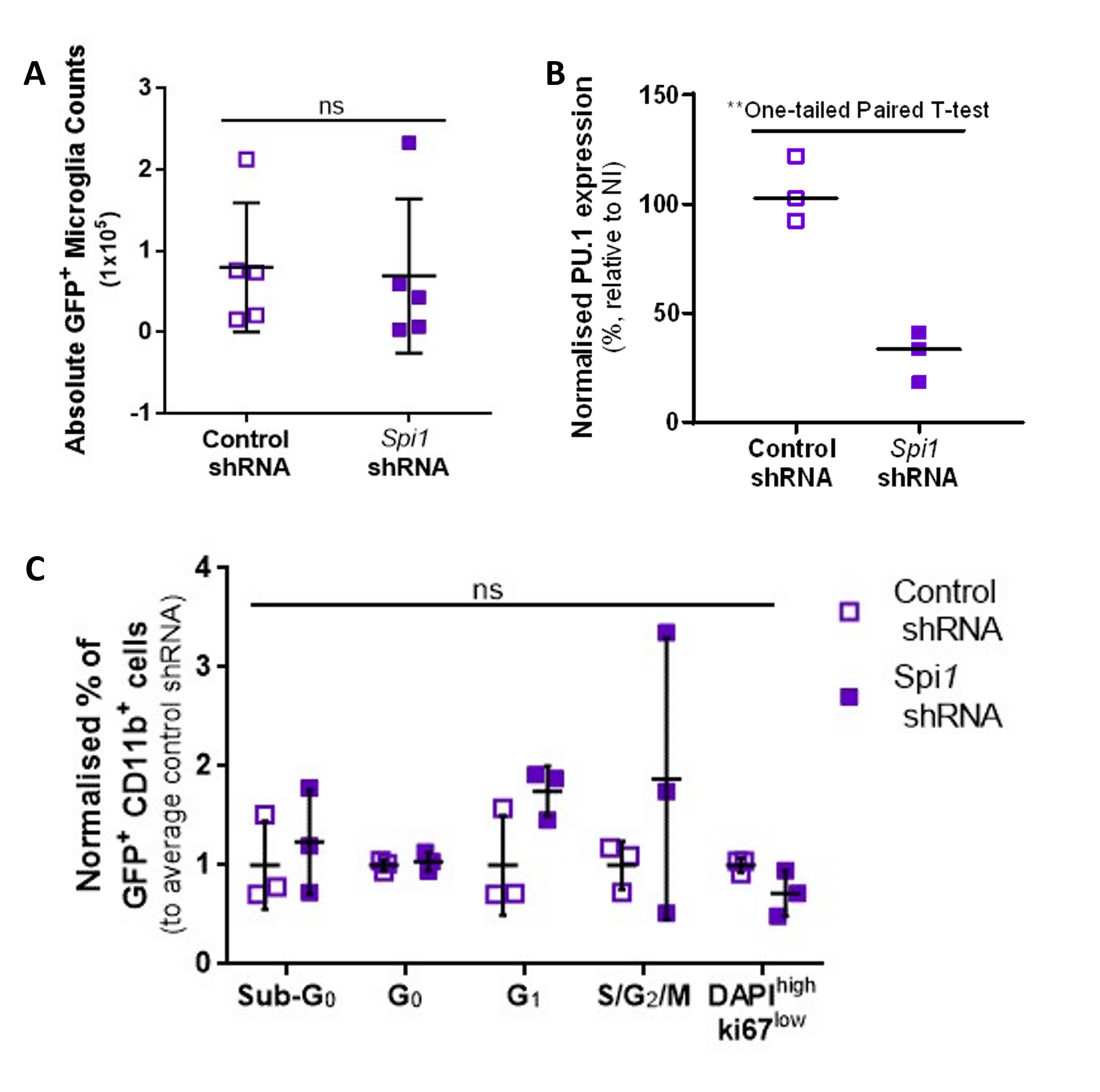


**Supplementary Figure 3- Functional validation of cell cycle alterations indicated by Spi1 knock-down RNA-Seq dataset. A** Absolute GFP+ microglia cell number did not differ between samples (One-tailed Paired T-test, p-value = 0.1456, n=5). **B** PU.1 expression measured in infected (GFP+) microglia (CD11b+) relative to a non-infected control. Again, Spi1 was significantly lower in Spi1 shRNA infected cells than in those that received the control shRNA (One-tailed Paired T-test, p-value = 0.001, n=3). **C** The percentage of events in each cell cycle phase was normalised to the average proportion of control shRNA infected CD11b^+^ cells. Spi1 dose had a significant contribution to the variation in fold-change between groups it did not significantly impact the phases of the cell cycle (Two-Way ANOVA P_Spi1Dose_ = 0.1202, P_CellCycle_ = 0.3406, P_int_ = 0.3406). Figures A and C were made in GraphPad PRISM 6 (version 3.07) and B in GraphPad PRISM 8 (version 8.4.3; both GraphPad Software, Inc.).


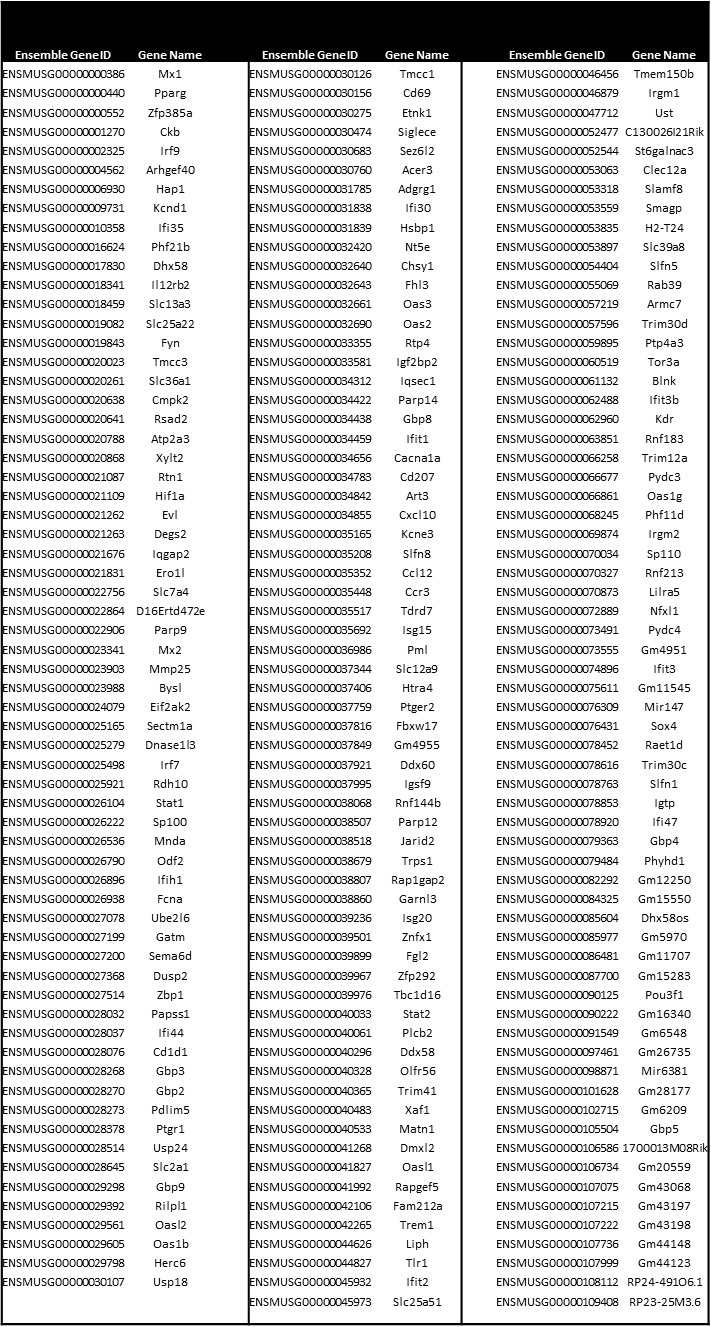


**Supplementary Table 1- 194 Spi1 dose-sensitive genes including Ensemble Gene ID and Gene Name.**


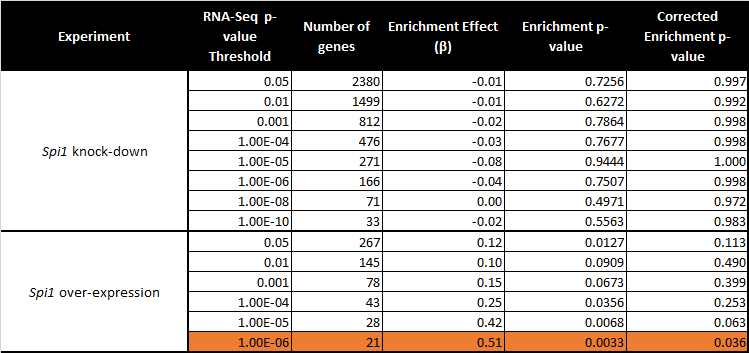
 **Supplementary Table 2- Information on the gene sets used in MAGMA analysis and the subsequent enrichment effects and p-values determined by this analysis.**


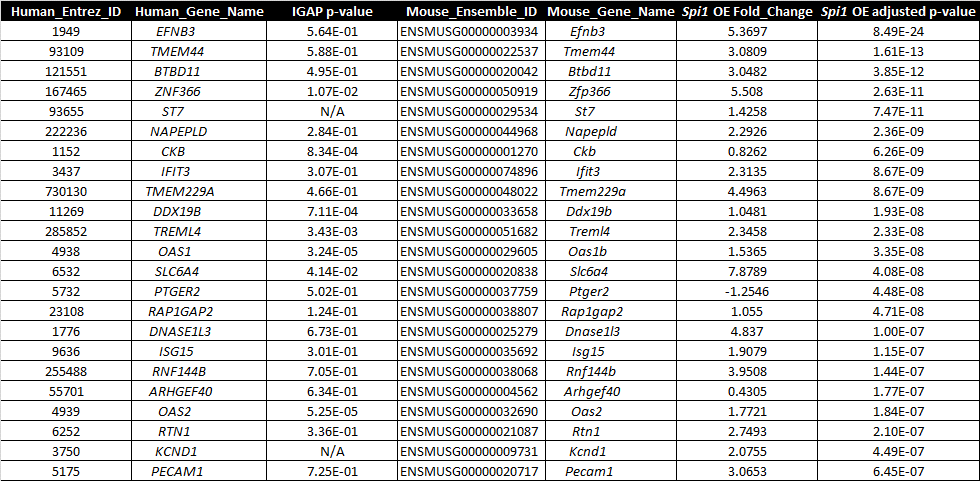


***Supplementary Table 3- P-values for the 21 Spi1 over-expression RNA-Seq genes that were significantly enriched in the IGAP dataset.***

library(gplots) # contains heatmap.2 function.

##
## Attaching package: 'gplots'

## The following object is masked from 'package:stats':
##
## lowess

library(dendextend) # make and color dendrogram.

##
## ---------------------
## Welcome to dendextend version 1.13.4
## Type citation('dendextend') for how to cite the package.
##
## Type browseVignettes(package = 'dendextend') for the package vignette.
## The github page is: https://github.com/talgalili/dendextend/
##
## Suggestions and bug-reports can be submitted at: https://github.com/talgalili/dendextend/issues
## Or contact: <tal.galili@gmail.com>
##
## To suppress this message use: suppressPackageStartupMessages(library(dendextend))
## ---------------------

##
## Attaching package: 'dendextend'

## The following object is masked from 'package:stats':
##
## cutree

library(colorspace)

#set working directory
setwd("C:/Users/rej66_000/OneDrive - Cardiff University/180626 RNA SEQ RESULTS/For Clustering")

#Load FPKM values with p-value <=0.05 as csv.
df <- read.csv("FPKM.0.05.csv")

#renamed rows with gene ID then remove ID column.
row.names(df) <-df$geneID
df <- df[,-1]

#Convert the dataframe into a data matrix, required for analysis.
dm <- data.matrix(df)

#Scaling the data, Log10 scale and scale data so the mean is 0.
Log_dm <- log10(dm+1)
SC_dm <- scale(Log_dm, scale = TRUE)
y <-SC_dm

#Hierarchical clustering of genes by row using pearson method, creating a dendrogram to show this.
dend1 <- as.dendrogram(hclust(as.dist(1-cor(t(y), method = "pearson")), method = "complete"))
c_group <- 6 #number of clusters data separated into.
dend1 <- color_branches(dend1, k = c_group, col = rainbow_hcl) #add color to the clusters in the dendrograms.
dend1 <- reorder(dend1, rowMeans(y, na.rm = T), agglo.FUN = mean) #re-order the data by the dendrogram into clusters.

#Take the cluster colours from the dendrogram and apply to the ordered cluster label.
col_labels <- get_leaves_branches_col(dend1)
col_labels <- col_labels[order(order.dendrogram(dend1))]

#Colours for heatmap, where blue indicates low expression and red high expression.
mycol <- colorpanel(75, "blue", "white", "red")

#Plot the heatmap with coloured dendrogram.
p <- heatmap.2(y, #scaled data matrix

 #Dendrogram and heatmap
 RowSideColors = col_labels,
 Rowv = dend1,
 Colv = FALSE,
 col = mycol,
 colRow = col_labels,
 density.info = "none",
 trace = "none",
 dendrogram = "row",

 #Key and labels
 key.title = "Scaled Z-Score",
 scale = "row",
 labRow = FALSE,
 cexCol = 1.2,
 labCol = c("NS shRNA 1", "NS shRNA 2","NS shRNA 3", "NS shRNA 4","Spi1 shRNA 1", "Spi1 shRNA 3", "Spi1 shRNA 4", "EV Control 1", "EV Control 2", "EV Control 3", "EV Control 4", "Spi1 OE 1", "Spi1 OE 2", "Spi1 OE 3", "Spi1 OE 4" ),
 offsetCol = -0.0000000001,
 margins = c(8,4),
 key = TRUE,

 #Creates a small space between sample sets
 colsep = c(4,7,11),
 sepcolor="white",
 sepwidth=c(0.05,0.05)
 )


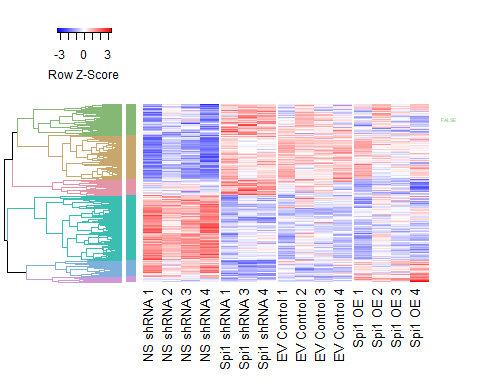


#Save cluster number against geneID
cluster <- as.matrix(col_labels)
output <- cbind(y, cluster)
write.csv(output, "R test Clustering output.csv", row.names = TRUE)

dev.off()

## null device
## 1

***Supplementary Figure 4 R Markdown for Clustering Analysis and generation of Figure 4a.*** *R Markdown was generated in R Studio* [6,7] *utilising code adapted from* [8,9]

## Supplementary Methods

### Validation of *Spi1* shRNA in RAW264.7 Cell Line

The *Spi1* shRNA was tested in the mouse macrophage RAW264.7 cell line which was maintained in DMEM media (containing 4.5 g/L D-Glucose, supplemented with 10 % (v/v) heat-inactivated Foetal Bovine Serum (FBS) and 100 units/mL Penicillin and 100 μg/mL Streptomycin; all Gibco), passaged twice weekly by gently scraping and maintained in a TC incubator at 37 °C with 5 % CO_2_. RAW264.7 cells were seeded at a density of 1x10^5^ per well of a 6-well plate (Greiner) and left to adhere overnight. The next day cells were infected with either control shRNA or *Spi1* shRNA and cultured for 1 week, media was changed as required. Cells were washed with PBS and harvested using Accumax (Sigma) at 37 °C for 10-20 minutes before wells were rinsed with DMEM media and centrifuged at 350 *x g* for 5 minutes.

The sample was then divided into two, where PU.1 expression was then assessed by flow (described in main methods) or sorted by GFP expression for *Spi1* mRNA assessment by qPCR.

Cells for qPCR analysis were then stained with LIVE/DEAD near-IR staining solution for 30 minutes on ice per manufacturers direction (Molecular Probes) and washed by centrifugation at 350 *x g* for 5 minutes in 0.5 % BSA (w/v), 5 mM EDTA in DPBS. Samples were re-suspended in 0.5 % BSA (w/v), 5 mM EDTA in DPBS and kept on ice before live GFP+ cells were collected on a fluorescence activated cell sorting, FACS Aria™ III (BD Biosciences).

The sorted cells were pelleted via centrifugation at 350 *x g* for 5 minutes, the supernatant aspirated and the pellet lysed for RNA using the Mini RNeasy kit (Qiagen) per manufacturer’s directions. RNA concentration was measured using the Nanodrop 2000 (Thermofisher Scientific). Per manufacturer’s direction between 0.5-1 μg of RNA was converted to cDNA by adding to 20 µL of the Precision™ Reverse-Transcription Premix 2 (PrimerDesign LTD) before being run in a Thermocycler (Eppendorf Mastercycler Nexus) at 42 °C for 20 minutes then at 72 °C for 10 minutes. Nuclease-free water was then added to make an equal total volume.

All qPCR reactions were performed with the 2X Precision FAST qPCR Master Mix (with SYBR green and low ROX, PrimerDesign LTD) per manufacturer’s directions. Per sample, 3 pM of the appropriate forward and reverse qPCR primers (sequences listed in **Supplementary Table 4**) were added to 5 µL master mix and 12.5 ng of cDNA, nuclease free water was used to give a reaction volume of 10 µL. All reactions were performed in triplicate and each plate (MicroAmp Fast Optical 96-Well Reaction Plates, Applied Biosystems) included the endogenous control gene *Ywhaz*. The plates were then sealed with a MicroAmp optical adhesive film and run on the Viia 7 Real-Time PCR system (Applied Biosystems) and assessed using the 40-ΔΔCT cycle method per the manufacturer’s guidelines using the QuantStudio Real-Time PCR software (version 1.3; Thermofisher Scientific) based on [10].


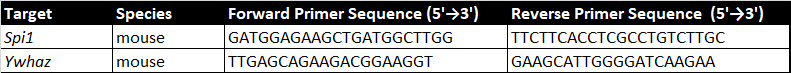


**Supplementary Table 4** qPCR primer sequences utilised to assess mouse Spi1 expression.

### Flow Cytometric Analysis of Ki67 Staining

Microglia were processed utilising a similar protocol that was flow cytometric analysis of PU.1, any differences are highlighted below. Briefly harvested cells were fixed in 1 % formaldehyde for 20 minutes on ice. Methanol permeabilization was not required as the wash solution was supplemented with 0.5 % Saponin. In addition to CD11b antibodies preciously mentioned, samples were stained with 2 μg/mL of ki67 PE-Cyanine 7 (SolA15) or Rat IgG2a,k isotype control (eBR2a, both eBioscience). Finally, 500 ng/mL of 4',6-Diamidino-2-Phenylindole Dilactate (DAPI; Thermofisher) was added to each sample and incubated for 20 minutes before flow cytometric analysis.

Supplementary Figure **5** demonstrates the gating strategy utilised to analysis the separate cell cycle populations in infected microglia (GFP^+^CD11b^+^). PU.1 expression was confirmed to be reduced in *Spi1* shRNA infected samples compared to control shRNA infected microglia (data not shown).


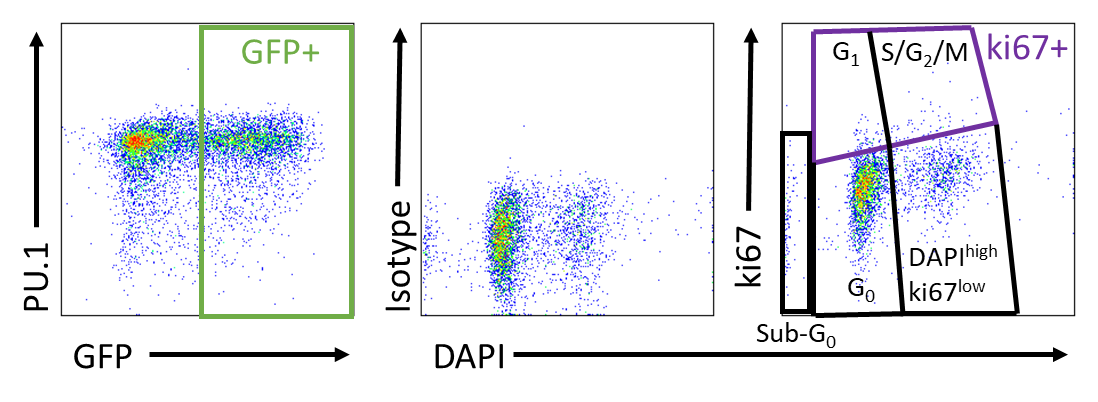


**Supplementary Figure 5 ki67 Gating Analysis-** GFP^+^ microglia (determined as CD11b^+^) were then divided into sub-populations of the cell cycle based on ki67 and DAPI staining as illustrated. In short, ki67^+^ cells were defined as having a higher MFI than isotype controls which were then further separated into G_1_ and S/G_2_/M based on DAPI staining. G_0_, DAPI^high^ cells were and sub-G_0_ cells were defined as ki67^-^ and separated according to the DAPI MFI.

Absolute GFP^+^ microglia number was calculated by dividing the percentage of GFP^+^ microglia (of all events) by 100 and multiplying this by the total viable cell number, determined by Muse Cell Analyzer (Merk Millipore) per manufacturer’s directions.

### Principle Component Analysis

Principle component analysis (PCA) was performed using the ‘prcomp’ function in R Studio (Version 1.2.5042 copyright 2009-2020 RStudio, Inc. [6]) with base R version 4.0.0 (2020-04-24, copyright 2020 The R Foundation for Statistical Computing [7]) with the help of the ‘tidyverse’ [1], ‘corrplot’ [2], ‘factoextra’[3], ‘rgl’ [4]and ‘plot3D’ [5] packages. A correlation matrix was generated from the FPKM normalised gene read counts reaching a significance threshold of P≤ 0.05, standardising the scores and giving them a mean of zero. The first three principle components (PC1, PC2 and PC3) accounted for 78 % of the variation between samples and were plotted using ‘plot3D’ package in R [5]. The loading scores for the top 10 genes contributing to PC1, PC2 and PC3 were also assessed.

## Supplementary References

1. Wickham H, Averick M, Bryan J, Chang W, McGowan L, François R, et al. Welcome to the Tidyverse. Journal of Open Source Software. 2019 Nov 21;4(43).

2. Wei Taiyun, Simko Viliam, Levy Michael, Xie Yihui, Jin Yan, Zemla Jeff, et al. R package “corrplot”: Visualization of a Correlation Matrix. 2021 [cited 2021 Jun 29]; Available from: https://github.com/taiyun/corrplot.

3. Kassambara Alboukadel, Mundt Fabian. factoextra: Extract and Visualize the Results of Multivariate Data Analyses [Internet]. 2020 [cited 2021 Jun 29]. Available from: https://cran.r-project.org/web/packages/factoextra/index.html

4. Murdoch Duncan, Adler Daniel, Nenadic Oleg, Urbanek Simon, Chen Ming, Gebhardt Albrecht, et al. 3D Visualization Using OpenGL [Internet]. 2021 [cited 2021 Jun 29]. Available from: https://cran.r-project.org/package=rgl

5. Soetaert K. plot3D: Plotting Multi-Dimensional Data [Internet]. cran R project; 2019. Available from: https://cran.r-project.org/web/packages/plot3D/plot3D.pdf

6. RStudio Team. RStudio: Integrated Development for R. . RStudio; 2020.

7. R Core Team. R: A Language and Environment for Statistical Computing [Internet]. R Foundation for Statistical Computing; 2020. Available from: https://www.R-project.org/

8. Liu Y. How to Draw Heatmap with Colorful Dendrogram [Internet]. Yang’s Research Blog. 2018 [cited 2020 Apr 23]. p. 1. Available from: https://liuyanguu.github.io/post/2018/07/16/how-to-draw-heatmap-with-colorful-dendrogram/

9. Brandon Yeo. How to plot a Heatmap in Rstudio, the easy way [Internet]. 2019 [cited 2020 Apr 23]. Available from: https://www.youtube.com/watch?v=OWWHfXgRw3k

10. Livak KJ, Schmittgen TD. Analysis of relative gene expression data using real-time quantitative PCR and the 2-ΔΔCT method. Methods. 2001;25(4):402–8.
